# Reducing visual deficits caused by refractive errors in school and preschool children: results of a pilot school program in the Andean region of Apurimac, Peru

**DOI:** 10.3402/gha.v7.22656

**Published:** 2014-02-13

**Authors:** Sergio Latorre-Arteaga, Diana Gil-González, Olga Enciso, Aoife Phelan, Ángel García-Muñoz, Johannes Kohler

**Affiliations:** 1 Entretodos Foundation, Zaragoza, Spain; 2 Public Health Research Group, University of Alicante, Alicante, Spain; 3 Department of Community Nursing, Preventive Medicine and Public Health, and History of Science, Faculty of Health Sciences, University of Alicante, Alicante, Spain; 4 CIBER Epidemiología y Salud Pública (CIBERESP), Spain; 5 Observatory of Public Policies and Health (OPPS), University of Alicante, Alicante, Spain; 6 Centro Oftalmológico Monseñor Enrique Pelach, Abancay, Peru; 7 Dublin Institute of Technology, Dublin, Ireland; 8 Departamento de Óptica, Farmacología y Anatomía, Universidad de Alicante, Alicante, Spain

**Keywords:** preschool children, primary school children, refractive errors, vision screening, teachers

## Abstract

**Background:**

Refractive error is defined as the inability of the eye to bring parallel rays of light into focus on the retina, resulting in nearsightedness (myopia), farsightedness (Hyperopia) or astigmatism. Uncorrected refractive error in children is associated with increased morbidity and reduced educational opportunities. Vision screening (VS) is a method for identifying children with visual impairment or eye conditions likely to lead to visual impairment.

**Objective:**

To analyze the utility of vision screening conducted by teachers and to contribute to a better estimation of the prevalence of childhood refractive errors in Apurimac, Peru.

**Design:**

A pilot vision screening program in preschool (Group I) and elementary school children (Group II) was conducted with the participation of 26 trained teachers. Children whose visual acuity was<6/9 [20/30] (Group I) and≤6/9 (Group II) in one or both eyes, measured with the Snellen Tumbling E chart at 6 m, were referred for a comprehensive eye exam. Specificity and positive predictive value to detect refractive error were calculated against clinical examination. Program assessment with participants was conducted to evaluate outcomes and procedures.

**Results:**

A total sample of 364 children aged 3–11 were screened; 45 children were examined at Centro Oftalmológico Monseñor Enrique Pelach (COMEP) Eye Hospital. Prevalence of refractive error was 6.2% (Group I) and 6.9% (Group II); specificity of teacher vision screening was 95.8% and 93.0%, while positive predictive value was 59.1% and 47.8% for each group, respectively. Aspects highlighted to improve the program included extending training, increasing parental involvement, and helping referred children to attend the hospital.

**Conclusion:**

Prevalence of refractive error in children is significant in the region. Vision screening performed by trained teachers is a valid intervention for early detection of refractive error, including screening of preschool children. Program sustainability and improvements in education and quality of life resulting from childhood vision screening require further research.

Refractive error can be defined as the inability of the eye to bring parallel rays of light into focus on the retina; instead of focusing images on the retina, the eye focuses light in front of the retina (myopia), behind it (hyperopia), or at two separate points near the retina, resulting in nearsightedness, farsightedness or astigmatism, respectively. Pediatric uncorrected refractive error is associated with increased morbidity and has extensive social and economic impacts, limiting educational opportunities and affecting subsequent quality of life in the adult population (1, 2). More than 12 million children aged 5–15 years old worldwide are visually impaired due to uncorrected refractive error (3), and it is the leading cause of visual impairment worldwide (4). The available data indicate that the incidence of refractive error is most frequent at 8–10 years of age (5). However, the timely diagnosis of refractive errors in children aged 3–5 years old remains an important goal because early treatment provides significant improvement of visual acuity and quality of life (6).

The case of myopia is especially striking due to its increasing prevalence worldwide in the last few decades. Although various research studies are currently underway and have contributed to a better understanding of the onset and progression of myopia, the regulatory process that causes refractive error still remains unknown; thus, prevention programs would appear as yet to be impracticable (7).

Vision screening is a method for identifying children with visual impairment or with eye conditions that are likely to lead to visual impairment so that a referral can be made to an appropriate eye care professional for further evaluation and treatment. Vision screening can be performed with the child's participation by subjectively testing visual acuity in children aged 3 years and older using age-appropriate vision acuity tests. The need to correct refractive error using optical compensation is determined by its effect on vision. Vision screening programs vary with regard to testing personnel, pass/fail criteria, methodology, frequency, and setting (8).

School has been recognized as the appropriate environment to strengthen health programs for prevention of disease and disability, and the need to continue building evidence and capturing practical experience in school health has previously been noted (9). Earlier reviews suggested that screening children for refractive error is economically attractive (5) and should be conducted at the community level and integrated into school health programs, accompanied by awareness campaigns to help remove the barriers to eliminating uncorrected refractive error (3).

The majority of previous studies with the explicit participation of teachers in vision screening programs show that they are able to perform basic vision tests adequately, although some considerations should be taken into account. Vision screening performed by teachers is less sensitive than a clinical examination performed by an eye specialist; reliability of vision screening is lower for those students who are already using optical correction; and it is advisable to inform parents that vision screening is not a diagnosis (10–14).

Peru's Apurimac region represents a vulnerable socioeconomic context with distinctive geographical and environmental conditions, such as high altitude and a rural lifestyle. According to 2009 census data, the Apurimac region has one of the highest poverty rates in the country (15). Access to eye care services is limited because there is a lack of specialized health-care personnel (16). Prevalence of uncorrected refractive error in the Andean region is not well known, although a previous cross-sectional study conducted in the 1990s in a neighboring location estimated prevalence rates of 7.8% for ocular pathologies in school children, including uncorrected refractive error. Prevalence of uncorrected refractive error was 4.6%, and astigmatism was the most prevalent vision disorder (3.2%). Females aged 5–9 years old were the most affected group (17).

With the aim of providing useful information on community-based eye health interventions, the objective of this study was to analyze the utility of a vision screening program conducted by teachers and to contribute to a better estimation of uncorrected refractive error prevalence among preschool and elementary school children in the area.

## Materials and methods

A pilot vision screening program initially conceived for 40 teachers was undertaken through the initiative of the COMEP Eye Hospital, located in Abancay, in the Apurimac region. A collaborative agreement was signed between COMEP and the educational authorities, the Dirección Regional de Educación de Apurímac (DREA), which defined the program commitments. One coordinator was designated in the eye health and educational institutions, respectively, to establish a permanent dialogue during the entire process. The study was planned, conducted, and evaluated in the period from September 2009 to June 2010.

### Training components

Teacher training was carried out in two sessions of four and a half hours each, outside school hours. A comprehensive visual health guide designed for teachers was compiled of knowledge and program procedures in Spanish; this was distributed among participant teachers. Instructions were offered for preschool and elementary schoolteachers, together with specific information for each age group. This approach was considered suitable because in this rural context it is frequent for children aged from 3 to 12 years old to be placed in the same class under the same teacher.

The training course was designed with the following modules and timing: I—Basic visual function and implications for the learning process (2 hours); II—Description of the most common vision disorders in children: signs and symptoms for detection (2 hours); III—Methodology for visual screening in the classroom (4 hours including supervised practice); IV—Healthy habits, risk prevention activities, and recommendations (30 min); and V—Pre- and post-questionnaire to evaluate the knowledge acquired (30 min).

The set of vision testing materials distributed included a Snellen optotype (tumbling E chart), a 6-m tape measure, an eye occluder, a penlight, data recording sheets, the schedule, and evaluation forms.

Teachers attending training were informed about the research nature of the intervention, and they performed the vision screening on students in the following days as an integral part of school activities. In a talk given by teachers in their respective schools, parental consent was sought prior to conducting the screening, for which instructions were given.

Normal visual acuity is commonly referred to as 20/20 (or even) vision; the metric equivalent is 6/6 vision. If a person has a visual acuity of 6/9 (20/30), he is said to see detail from 6 m (20 feet) away the same as a person with normal eyesight would see it from 9 m (30 feet) away. Visual acuity refers to acuteness or clearness of vision, which is dependent on the sharpness of the retinal focus within the eye and the sensitivity of the interpretative faculty of the brain (18).

The battery of vision screening tests conducted by teachers consisted of testing each child's presenting visual acuity using the tumbling E chart at a distance of 6 m, first monocularly and then binocularly. This was followed by checking pupil reaction to direct light while at the same time detecting visible opacities affecting corneal transparency. When giving instructions on observation, participants were informed that direct contact with the fingers was unnecessary. Presenting visual acuity at 6 meters for one or both eyes that was less than 6/9 (20/30) for preschool children and less than or equal to 6/9 (20/30) for elementary school children was considered the cutoff point for referral.

The presence of nonreactive pupils (when exposed to direct light at 10–15 cm from each eye) was also indicated as a direct cause for referral. A teacher's time to perform vision screening in the classroom was estimated to be 5–10 min per child. Children who did not meet the screening criteria were referred to the COMEP Eye Hospital for a comprehensive eye examination. In each case, treatment, diagnosis, and recommendations were properly explained to parents or guardians.

### Sampling strategy

In 2010, the ministry of education calculated that the school population in Abancay province aged 3–11 years old was 21,566. To obtain a sufficient sample, we considered a refractive error prevalence of 6% and assumed an accuracy of 3% with a confidence interval (CI) of 95%. Based on these figures, the minimum sample size was estimated at 239 schoolchildren. In our study, participant schools were selected by educational authorities; the schoolchildren samples were selected from whole classes to be screened by their usual teacher (who received the training).

### Prior validation of concordance

In order to estimate the validity of vision screening performed by teachers, two ophthalmic assistants from the COMEP clinical team—who conducted visual acuity testing in their daily practice—visited randomly selected schools to repeat in situ vision screening in 63 children (15% of the total sample) under the same conditions of space and lighting. The concordance of results, particularly with regard to the visual acuity test, was analyzed separately for the two age groups.

### Clinical examination in referred children

Eye examination of referred children was performed in the COMEP hospital to confirm the presence of uncorrected refractive error. Medical histories were obtained in the presence of parents or guardians. The refractive examination included slit lamp examination, autorefraction with keratometry (using Nidek ARK-700A), and retinoscopy. Refraction in cycloplegia was performed when necessary. The Titmus Fly Stereo Test and cover-uncover test were used to assess binocular function. Children presenting with other pathologies were subsequently referred for a full medical examination. In each case, costs for the necessary treatment were fully or partially subsidized by the hospital.

Prescription for refractive error was indicated when uncorrected distance visual acuity was significantly reduced. In our study, we considered significant refractive error as myopia>−1.00; prescription for astigmatism and hyperopia was determined according to presenting vision reduction, symmetry of refractive error between both eyes and binocular function improvements according to age. Hyperopia>+3.00 and astigmatism>1.50 were considered a reference for minimum optical correction (19); age was also a determining factor in establishing the final prescription. In the presence of no significant refractive error, a checkup was recommended after 6 months. Benefits and outcomes of continuous access to eye care services are not included in the scope of this study.

## Data analysis

Positive predictive value (PPV) for the vision screening performed by teachers to detect uncorrected refractive error and for the capacity to correctly classify children free of refractive error, defined as specificity, were calculated against clinical examination based on validated results of concordance and were estimated on the whole sample.

An epidemiological analysis was conducted on the data obtained to calculate 95% CI using the free software package, Epidat version 3.1 (20). Referred children who failed to attend clinical appointments were excluded in the correlation analysis and in the prevalence estimations of uncorrected refractive error. Prior validation of concordance was obtained through direct comparison at school of visual acuity results obtained by teachers and by ophthalmic assistants for the same children.

Teachers’ perspectives on aspects of the training were considered a program outcome and were collected through an anonymous questionnaire. Several statements were presented, and participants were asked to indicate their level of agreement on a scale from 0 to 4 (strongly disagree to strongly agree). Results were calculated as a unit average score.

At the closing event conducted by health and education coordinators, a feedback meeting was scheduled with participant teachers. Pilot program outcomes were presented, and potential improvements to the process were openly discussed.

## Results

Twenty-six teachers attended the first training season and completed the process. When compiling files, five reports from teachers were erroneous or incomplete and therefore not included in the study. A sample of 364 children aged 3–11 years old taught by 21 teachers in 19 preschool and elementary school institutions was finally eligible for inclusion to verify consistency of vision screening performed by teachers.

Prevalence of uncorrected refractive error in this population was found to be more than 6%. The data obtained are presented in Table 1 by group (Group I: preschool children and Group II: elementary school children). Sixty-eight children were referred by teachers, and a final sample of 45 children (66%) was examined at the eye hospital where significant uncorrected refractive error was confirmed in 24 children. Astigmatism and hyperopia combined with astigmatism were observed as the most common refractive errors. Findings of refractive error and eye disorders were not categorized in detail because this study focused on procedure and on teachers’ efficiency as screening agents.

**Table 1:** Auditing process of vision screening outcomes and understanding reached by participants

	Teachers (n)			Children (n)			Clinical findings		
			
	Invited	Trained	Completed report	Screened	Referred	Attended	N	RE	Prevalence CI 95%
Group 1 (aged 3–5)	20	14	11	213	31	22	204	13	6.2 [2.82–9.52]
Group 2 (aged 5–11)	20	12	10	174	37	23	160	11	6.9 [2.64–11.11]
Monitor:	Planning>		Teacher performance		>Access		>Prevalence of URE		
			Perspectives						
	
			Coordinators				Teacher participants		
• R1: 35% of invited teachers did not attend training			- Reinforce communication channels between teachers and coordinators. - Motivate teachers to attend to their own visual needs as some of them may need an eye examination.				- Consider school schedule to avoid overlapping with other academic events. - Acknowledge teacher participation with a certificate to reinforce curriculum. - Confirm attendance in advance.		
• R2: 20% of files provided were incomplete or contained errors			- Methodology and data management deserves more attention in the training process. - Simplify the design to avoid irrelevant data and focus on fundamental information: visual acuity results, pupil findings, and parental information.				- Extend the duration of theoretical and practical training.		
• R3: One in three referred children did not attend their clinical eye examination.			- Facilitate attendance at the eye hospital by considering this when planning the program and estimating the budget. - Confirmation of parents having been informed.				- Provide shared transport to lower costs. - Invite proactive parents to attend the training, in order to raise awareness in the community. - Include study activities as part of the program to encourage the use of eyeglasses and promote their acceptance among children.		

RE, refractive error; URE, uncorrected refractive error; R1, R2, R3, results discussed.

Specificity of vision screening by teachers after clinical examination in Groups I and II was estimated at 95.8% and 93.0%, respectively, while PPV was 59.1% and 47.8%, respectively; results with a 95% CI are shown in Table 2. No case of nonreactive pupils to direct light was reported by teachers. It is worth mentioning that, when the case history was taken, none of the children examined reported ever having undergone a clinical eye examination before. Slight allergenic irritations and mild conjunctivitis were found in around one in four children examined at the Eye Hospital. Distribution of vitamin A capsules and promotion of healthy nutritional habits and risk prevention—such as washing hands and avoiding exposure to dust, smoke, or excessive direct sunlight—were recommended by an experienced nurse familiar with the rural context.

**Table 2:** Results of vision screening performed by teachers

	Group 1: aged 3–5		Group 2: aged 5–11	
		
	%	CI 95%	%	CI 95%
Specificity	95.8	92.8–98.7	93.0	89.0–96.9
PPV	59.1	36.3–81.9	47.8	25.2–70.4

CI, confidence interval; PPV, positive predictive value.

When assessing concordance in the visual acuity test, the results obtained by ophthalmic assistants were recorded and compared to those obtained previously by teachers in a sample of 63 children. Our findings are as follows:

1. In a sample of 33 children in Group I (children aged 3–5), better visual acuity results (6/6) in at least one line in one eye were obtained in 15 cases (45%) by ophthalmic assistants. In all other cases, the results coincided.
2. In a sample of 30 children in Group II (children aged 6–11), better visual acuity results were obtained in two cases (7%) by ophthalmic assistants. In the remaining 28 cases (93%), the visual acuity results coincided.
3. In no case in either group were the visual acuity results lower than those registered earlier by teachers. No visual acuity deficits passed unnoticed by trained teachers when performing vision screening.

In the questionnaire completed by teachers once the screening was performed, the level of agreement scores (from 0 to 4) obtained were: 1) usefulness of program contents for required teaching competencies: 3.8; 2) suitability of duration of theoretical and practical training from the teachers’ point of view: 1.4 and 1.2, respectively; 3) parental interest in the school-based vision program: 3.0; and 4) collaboration of children when performing Vision Screening in the classroom: 3.4.

In the feedback meeting with participants, proposals were presented after a discussion of outcomes and in consideration of coordinators’ and teachers’ perspectives (Table 1). The main recommendations were to prolong the duration of training, to increase awareness by inviting parent representatives from each school to training, and to take the travel distance required of referred children into consideration when planning and estimating the budget for the program.

## Discussion

Our findings show that uncorrected refractive errors in the preschool and school age population are a significant public health issue in this region. Trained teachers constitute valid screening agents for identifying deficits in visual acuity according to age-related expectations, including children aged 3–5 years old. Individual and group assessment by participants was mostly positive, and they made proposals for improving the organization and effectiveness of the intervention.

Regarding the limitations of this study, it is important to note that the estimation of uncorrected refractive error prevalence in school programs may not represent reality because official data on the percentage of children attending school in the Apurimac region is 75% and 94% for children aged 3–5 and 5–11, respectively (21). Therefore, the school population may not coincide with the total child population. Moreover, in the poorest countries, access to education by children with disabilities may fall to rates of 10% (22), and this may be addressed by strategies that aim to improve universal education (12). With regard to the procedures applied in the intervention, the use of visual acuity measurements for detecting significant refractive errors in children may not always be effective for detecting hyperopia and /or astigmatism (23).

Teacher attendance at training (65% of the initial estimate) indicates an unnecessary loss of efficiency in the use of resources. However, our final number of trained teachers was within the range reported in other programs (11, 14) and remains sufficiently large to draw conclusions as expected in a pilot intervention.

Of the children referred by teachers, 66% of the expected number attended the clinical appointment. Failure to attend has also been reported in studies conducted in India (12) and Iran (24) and generally is related to difficulties parents face in assuming the travel costs and other factors in their daily lives. In our study, increasing levels of effort and investment to raise awareness and to support parents were highlighted as factors for improving access to eye care services. In our case, in terms of sustainability, the complete elimination of economic barriers such as the suitable frames and adequate lenses for children is a concern that remains unresolved.

Different studies that have achieved optimal results in specificity or sensitivity associate these results with factors such as age at testing, specialization or training of those conducting the screening (12, 25), the experience accumulated by teachers who perform vision screening annually with health staff monitoring (24), and a more permissive visual acuity cutoff criteria such as 6/18, which only detects children below official vision impairment thresholds (26). This is in contrast to other studies that have suggested that a visual acuity cutoff point of less than 6/9 will miss significant refractive error that should not be ignored because this may have consequences for the learning process (27). In our study, we consider that a visual acuity of less than 6/9 for children aged 3–5 would be a more efficient criterion to avoid unnecessary referrals. When analyzing the correlation between visual acuity measured by teachers and subsequently by visiting ophthalmic assistants, it was observed that a significant number of preschool children (45% of cases) presented better visual acuity when measured by the latter. A plausible explanation for this, particularly in this age range, is related to the child's greater familiarity with the test when it is repeated shortly afterward. However, these findings do not affect the validity of visual screening because they do not represent false negatives but rather a slight improvement in visual acuity for children whose vision was already within acceptable limits.

The PPV of vision screening performed by teachers to identify uncorrected refractive error may be improved because it was low for both groups but particularly among children aged 5–11 years old (47.8%). Along with revisiting training methods, an increase in the time expended on teacher training, in the theoretical portion and in performing visual acuity tests, was considered a positive move to achieve a better predictive value. In response to these recommendations, participants’ suggestions were included in a reedited version of the training guide that was made available to the public in May 2011 (28). However, the benefits of adapting training materials and implementing such recommendations are not discussed in this article.

We conceived the school eye health program as a shared responsibility between educational and health institutions and in accordance with the interests of educators who want to see improvements in accessibility, coverage, and school performance. In addition, teachers have proven to be influential agents in promoting community health through the dissemination of information about healthy habits and risk prevention and by demystifying misconceptions. The commitment of teachers who have a better understanding of the barriers produced by visual disorders may enable them not only to perform a rudimentary eye test but also to develop practical skills such as recognizing signs of eyestrain, correcting postural errors, or adapting working distances when subjects with visual limitations are identified.

The 2013 State of the World's Children Report, Children with Disabilities, calls for an end to discrimination against children with disabilities and also for a focus on their abilities and potential instead of what they cannot do (29). School-based eye health programs contribute to eliminating barriers to children with undetected vision deficiency, and vision screening is the first step to ensuring that students engage in the learning process in optimal visual conditions. Integration of visual health skills into teaching competencies can contribute to improving vision screening programs. Moreover, community health advocacy in which teachers play an active role provides added values aligned with the goals of promoting the school as a healthy environment (30). Furthermore, early child development and education are determinants of living and working conditions in adulthood, and they should be given priority in international strategies and policies (31).

## Conclusions

Prevalence of refractive errors in children aged 3–12 is significant in the region. The coordinated intervention of trained teachers together with specialized eye care services provides a valuable opportunity to contribute to the prevention of correctable childhood visual deficits. Trained teachers have shown optimal validity for the early detection of visual acuity deficits caused by refractive problems, even in preschool children. Two fundamental aspects of the program were to engage the teachers and to seek their opinions on this health intervention. Further research is required in order to ensure sustainability and accurate procedures of vision screening programs as well as to measure improvements to the quality of life or other economic impacts resulting from childhood vision screening.
